# Periurethral Smooth Muscle Tumor of Undetermined Malignant Potential

**DOI:** 10.1155/2012/546852

**Published:** 2012-06-05

**Authors:** Jessica Hsieh, Sarah Collins, Christopher M. Morosky

**Affiliations:** ^1^Department of Obstetrics and Gynecology, University of Connecticut Integrated Residency Program, 263 Farmington Avenue, Farmington, CT 06030-2947, USA; ^2^Urogynecology Division, Department of Obstetrics and Gynecology, University of Connecticut Integrated Residency Program, Farmington, CT 06030-2947, USA

## Abstract

Smooth muscle tumors of undermined malignant potential (STUMP) are atypical smooth muscle tumors. The majority of these tumors are of uterine origin. We report the first known periurethral STUMP. Complete surgical resection is recommended for all cases of STUMP. They can recur in the form of STUMP or leiomyosarcoma.

## 1. Introduction

Smooth muscle tumors of undetermined malignant potential (STUMP) are rare smooth muscle tumors that are not definitively benign or malignant on histologic evaluation [1]. The majority of STUMPs are of uterine origin. In this paper, we present the first known case of a periurethral STUMP that was treated by surgical excision.

## 2. Case Report

**Figure 1:**
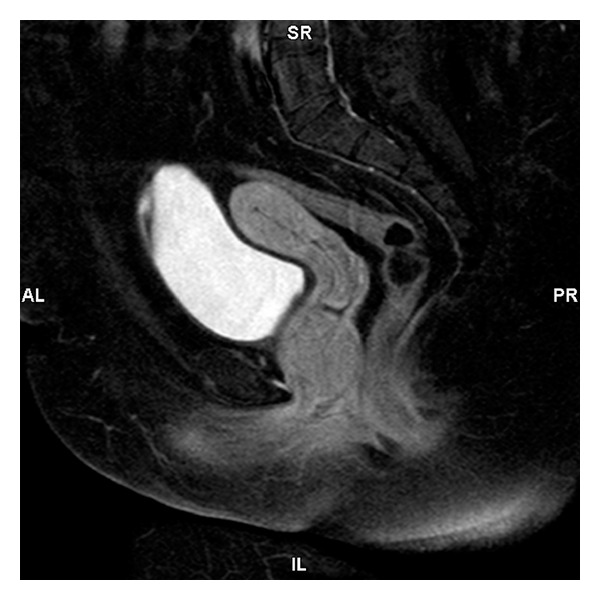
T2-weighted MRI of the pelvic characterized a 4.6 × 4.2 × 4.0 centimeter mass located within the anterior vaginal wall with mild homogeneous enhancement.

A 37-year-old nulligravid female presented to our clinic for an annual gynecologic examination. There were no abnormal findings noted on pelvic exam and a screening Pap smear was performed. Four weeks later, the patient returned for a colposcopic examination for findings of low-grade squamous intraepithelial lesion on her Pap smear. She was without complaint at this follow-up visit. However, her cervix was obstructed from view by a new anterior vaginal wall mass. The patient was sent for voiding cystourethrogram (VCUG), which did not demonstrate a urethral diverticulum. Subsequently, the patient was sent for magnetic resonance imaging (MRI). The MRI characterized a 4.6 × 4.2 × 4.0 centimeter mass located within the anterior vaginal wall with mild homogeneous enhancement (Figure 1). After consultation with a urogynecology specialist, the patient was taken to the operating theater for excision of the mass via the vaginal approach. A transverse incision was made along the anterior vaginal wall epithelium overlying the periurethral mass. Two solid individual masses were then carefully dissected away from the urethra. The periurethral tissues were closed in layers using a pants-over-suit technique.

**Figure 2:**
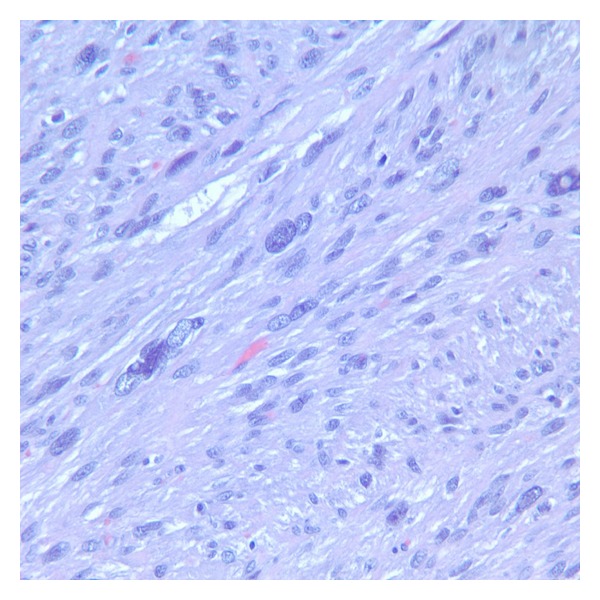
Hematoxylin and eosin stain of periurethral STUMP tissue showing diffuse moderate atypia.

A gross inspection by the surgeons revealed two soft, tan, smooth masses with no evidence of necrosis upon cross-section. They were felt to be periurethral leiomyomas and were sent to pathology for permanent section. The dimensions of the masses were 4.3 × 3.5 × 3.5 centimeters and 4.0 × 3.7 × 2.5 centimeters. Final histopathology revealed smooth muscle tumors with diffuse moderate atypia, absent necrosis, and less than 2 mitotic figures (MFs) per 10 high powered fields (HPFs) (Figure 2). The final diagnosis was STUMP. A second, independent pathologist was consulted and agreed.

## 3. Discussion

Three recognized features of malignant smooth muscle tumors are moderate-to-severe cytologic atypia, a mitotic count of ≥10 MF per 10 HPF, and tumor cell necrosis. If a tumor is clinically malignant with 2 of the 3 features, it is diagnosed as a leiomyosarcoma [2]. In contrast, benign leiomyomas are defined as smooth muscle tumors with no atypia ≤4 MF per 10 HPF and no tumor cell necrosis. Smooth muscle tumors that do not fit into these categories are diagnosed as STUMP. Most STUMPs are of uterine origin. In our literature search, there had only been one other case of extrauterine STUMP, describing a vulvar STUMP with local recurrence in a 10-year-old patient [3]. It is unclear whether extrauterine STUMPs have different clinical courses when compared to those of uterine origin. The diagnosis of periurethral STUMP in our patient represents an extremely rare occurrence of extrauterine STUMP. This is the first report known to these authors of STUMP involving the anterior vaginal wall. It is difficult to predict the clinical outcome for this patient, as the clinical courses of uterine STUMP are widely variable. In a retrospective review of 41 cases of uterine STUMP, there was an overall 7 percent recurrence rate in the form of both STUMP and leiomyosarcoma [4].

There is no consensus for the clinical management of patients with STUMP. Complete surgical resection is the initial treatment. Currently there is no role for adjuvant chemotherapy or radiation therapy following initial surgical treatment. Due to the possibility of recurrence and malignant transformation of STUMP, these patients require followup more frequently than annual visits. In our case, we plan to follow our patient every 3 months with pelvic exams for two years, saving MRI for any suspected recurrence. Future studies are needed to propose recommendations for the clinical management of extrauterine STUMP.

## 4. Disclosure

None of the authors have a conflict of interest.

## References

[1] Devilee TF (2003). *World Health Organization Classification of Tumours: Pathology and Genetics of Tumours of the Breast and Female Genital Organs*.

[2] Bell SW, Kempson RL, Hendrickson MR (1994). Problematic uterine smooth muscle neoplasms: a clinicopathologic study of 213 cases. *The American Journal of Surgical Pathology*.

[3] Harrington A, Bell E, Suchi M, Behmaram B, Uyar D (2011). Multifocal vulvar smooth muscle tumor with an unusual intravascular growth pattern and multiple local recurrences in a 10-year-old child: a diagnostic dilemma. *Pediatric and Developmental Pathology*.

[4] Guntupalli SR, Ramirez PT, Anderson ML, Milam MR, Bodurka DC, Malpica A (2009). Uterine smooth muscle tumor of uncertain malignant potential: a retrospective analysis. *Gynecologic Oncology*.

